# The effects of stroke on weight transfer before voluntary lateral and forward steps

**DOI:** 10.3389/fneur.2022.891439

**Published:** 2022-07-22

**Authors:** Marcel Bahia Lanza, Vicki L. Gray

**Affiliations:** Department of Physical Therapy and Rehabilitation Science, University of Maryland School of Medicine, Baltimore, MD, United States

**Keywords:** stroke, stepping, lateral, forward, weight transfer, hip abductors, torque, balance

## Abstract

There is a higher rate of falls in the first year after a stroke, and the ability to step in different directions is essential for avoiding a fall and navigating small spaces where falls commonly occur. The lateral transfer of weight is important for stabilizing the body before initiating a step. Hence, understanding the ability to control lateral weight transfer (WT) in different step directions might help understand falls in individuals with stroke. The present study aimed to compare the WT characteristics (onset time, duration, mediolateral center of pressure (ML COP) velocity, and ML COP displacement) and hip abduction torque preceding a lateral and forward voluntary step between individuals with stroke (paretic and non-paretic leg) and controls. Twenty individuals with stroke and ten controls performed voluntary choice reaction tests in the lateral and forward directions. Ten trials (five on each side—right and left) were performed for each step direction. The overall primary findings were that (1) the WT before a lateral step was shorter and initiated earlier, with a larger ML COP displacement and greater hip abductor torque in the stepping leg than the forward step, (2) there was greater hip abductor produced in the stance leg before a forward step than a lateral step, (3) the WT before the lateral step took longer to initiate and was slower to execute in individuals with stroke regardless of the leg (4) the WT before the forward step had more differences in the paretic than the non-paretic leg. Thus, for the first time, it was shown that the WT characteristics and hip abduction torque during the WT are different according to step direction and also appear to be impaired in individuals with stroke. These results have implications for understanding the direction that individuals with stroke are more susceptible to being unable to recover balance and are at risk of falling.

## Introduction

There is an approximately 40% chance of falling in the first year after a stroke (1). A common strategy to avoid falling is to take a protective step quickly to recover balance (2) in the direction of the instability. The ability to step in different directions (e.g., lateral or forward) is essential for avoiding a fall and navigating small spaces, such as the home, where falls commonly occur (3, 4). Regardless of the step direction, a lateral weight transfer (WT) to the stance (or supporting) leg precedes a step. The lateral transfer of weight is important for stabilizing the body before initiating a step (5–7). Understanding the ability to control lateral WT in different step directions may be necessary for understanding falls in individuals with stroke.

Performing a voluntary step as quickly as possible is a strategy that may prevent a fall (7). In older adults, the response time can distinguish fallers from non-fallers (8). After a stroke, the ability to perform a voluntary step is more impaired than age-match controls (2, 9), with individuals with stroke taking longer to initiate WT (9) and executing a voluntary step slower than older controls (10). The differences in step performance might be due to impaired WT characteristics. Older adults have a WT duration ranging from 134 ms (6) to 207 ms (7). In comparison, individuals with stroke take a longer time to WT [~380 ms (non-paretic leg) to ~420 ms (parectic leg)] while walking (e.g., forward stepping) (11). Nonetheless, individuals with stroke might be slower due to the asymmetric weight-bearing that compensates for the sensory and motor deficits in the paretic leg in individuals with stroke (12). Notably, the abovementioned studies have investigated different tasks (forward voluntary step vs. walking), which could influence the transfer of weight preceding the task. Thus, it would be important to determine whether WT differs between step directions (lateral vs. forward step) and whether the WT differs from controls.

The capacity to produce hip abductor torque influences the ability to step (7, 13, 14). The WT, partially controlled by the ability of the hip abductors to produce torque (7), plays an essential role in balance and mobility across ages (15). Older adults who produce an earlier and greater hip abductor torque in the stance leg (in the first 300 ms) during a maximal contraction transfer weight faster prior to a voluntary lateral step (7, 16). Thus, rapidly producing torque with the hip abductor muscles might help individuals take a step with the appropriate temporal and spatial characteristics to avoid a fall. Although, most studies do not examine torque production during the task but rather in a single joint maximum contraction. Nonetheless, individuals with stroke have an interdependence of interlimb (non-paretic vs. paretic leg) function (stance vs. stepping leg) during a voluntary step (2). Additionally, with a decrease in function of the paretic leg, this interdependence impairs the ability to step when the paretic leg acts as a stance or stepping leg (9, 17, 18). Hence, the ability to generate a quick and earlier abductor torque may impact the WT before a voluntary step depending on the leg used (non-paretic vs. paretic leg) in individuals with stroke. Thus, assessing the hip abductor torque production in-task (while stepping) is important. Furthermore, determining the difference in WT based on step direction, and the difference between individuals with stroke and a group of controls of a similar age may help understand falls in individuals with stroke.

Therefore, the present study aimed to compare WT characteristics (onset time, duration, mediolateral center of pressure displacement and center of pressure velocity) and hip abduction torque preceding a lateral and forward voluntary step between individuals with stroke (paretic and non-paretic leg) and controls. We hypothesized individuals with stroke would have impaired WT characteristics and reduced hip abductor torque (paretic and non-paretic legs) in both step directions (lateral vs. forward) compared to controls, with the paretic-leg being more impaired compared to the non-paretic leg.

## Material and methods

### Subjects

Power analysis was performed for the repeated measures analysis of variance (ANOVA) with a between factor and calculation was performed by the software GPower (version 3.1) (19), and the following inputs were used: (a) Effect size f (V) = 0.65; effect size was estimated by partial eta squared of 0.3; (b) alpha = 0.05; (c) Power = 0.8; (d) Number of groups = 3 (paretic x non-paretic x controls); and (e) Number of measurements (lateral x forward step) 2. A total sample size of 21 participants was required to achieve significance. Twenty community-dwelling adults with hemiparesis and ten controls were recruited for this study. For the inclusion criteria, participants were included if they were >6 months post-stroke, ≥ 50 years of age, could stand unsupported for 5 min, were able to walk 10 m with or without an assistive device, and did not have a medical condition that significantly impacted their ability to walk beyond the effects of the stroke. For the control group, participants were included if they had no self-reported history of a neurological injury or condition. All subjects provided written informed consent. Experimental procedures were approved by the University of Maryland, Baltimore Institutional Review Board.

### Procedures

Participants attended one testing session and performed a stepping assessment and a clinical assessment of balance and motor recovery.

#### Stepping assessment

Participants performed a block of ten lateral and ten forward choice reaction steps (CRT). For the lateral and forward steps, participants wore a safety harness and stood in their comfortable stance width on two adjacent force platforms (Advanced Mechanical Technology Inc., Watertown, MA, USA). The outline of their feet was traced on a piece of paper taped to the force platform to ensure the foot placement was similar for each trial. A metal pole with a horizontal bar positioned at eye level containing a light at the right and left end of the horizontal bar was positioned 6 feet directly in front of the subject. The light cue indicated to take a step in the direction of the light (i.e., right light right step). Participants were instructed to take a step as fast as they could following a visual “go” cue” without prior knowledge of the stepping leg [5 trials x 2 sides (five paretic/non-paretic or left/right for the individuals with stroke/controls)]. Steps were performed in blocks, with all steps from the lateral step being performed first, followed by the forward steps. A break of at least 5 min was provided between blocks.

#### Recording

The kinematic and kinetic data were sampled at 120 Hz and 600 Hz and collected for 7 s. Reflective markers were placed on the foot, ankle, hips, shoulders, and head, creating a seven segment skeletal model. The kinematic data were recorded using a 10-camera motion analysis system (Vicon, Oxford, UK) (9). The signals were smoothed using a four-order Butterworth filter with a cut-off frequency of 8 Hz. The ground reaction forces were filtered with a 10 Hz cut-off frequency. The vertical ground reaction forces were monitored visually by an investigator to ensure symmetrical weight-bearing before the start of each trial. The participants were instructed to evenly distribute their weight only when asymmetry was observed in the ground reaction forces.

#### Clinical assessment

Clinical tests of balance and balance confidence were assessed with the Timed Up and Go (TUG), Activities-specific Balance Confidence (ABC), and Community Balance & Mobility (CB&M). These measures are validated in individuals with stroke (20, 21). The Chedoke McMaster Stroke Assessment Impairment Inventory (CMSA) was used to assess the leg and foot motor recovery in the stroke group. The CMSA stages the level of motor recovery with the stages of motor recovery graded from 1–7, with 7 being classified as normal (22). The cutaneous sensation of the plantar aspect of the foot was assessed by a series of Semmes-Weinstein monofilaments, ranging from 1.65–6.65, with the lowest value representing normal cutaneous sensation.

### Data analysis

**Figure 1:**
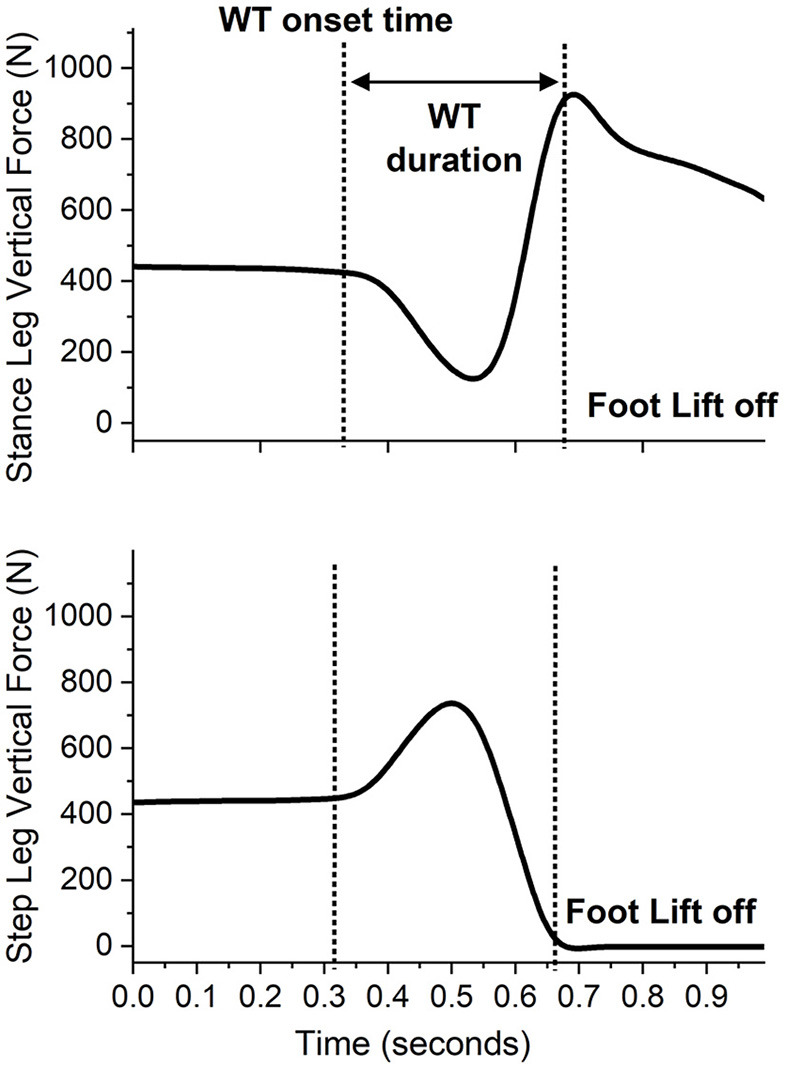
A sample recording of the vertical ground reaction forces during a single forward step of an individual with stroke stepping with the paretic leg while the non-paretic leg is the stance limb. The presentation of the light cue is at time 0.

**Figure 2:**
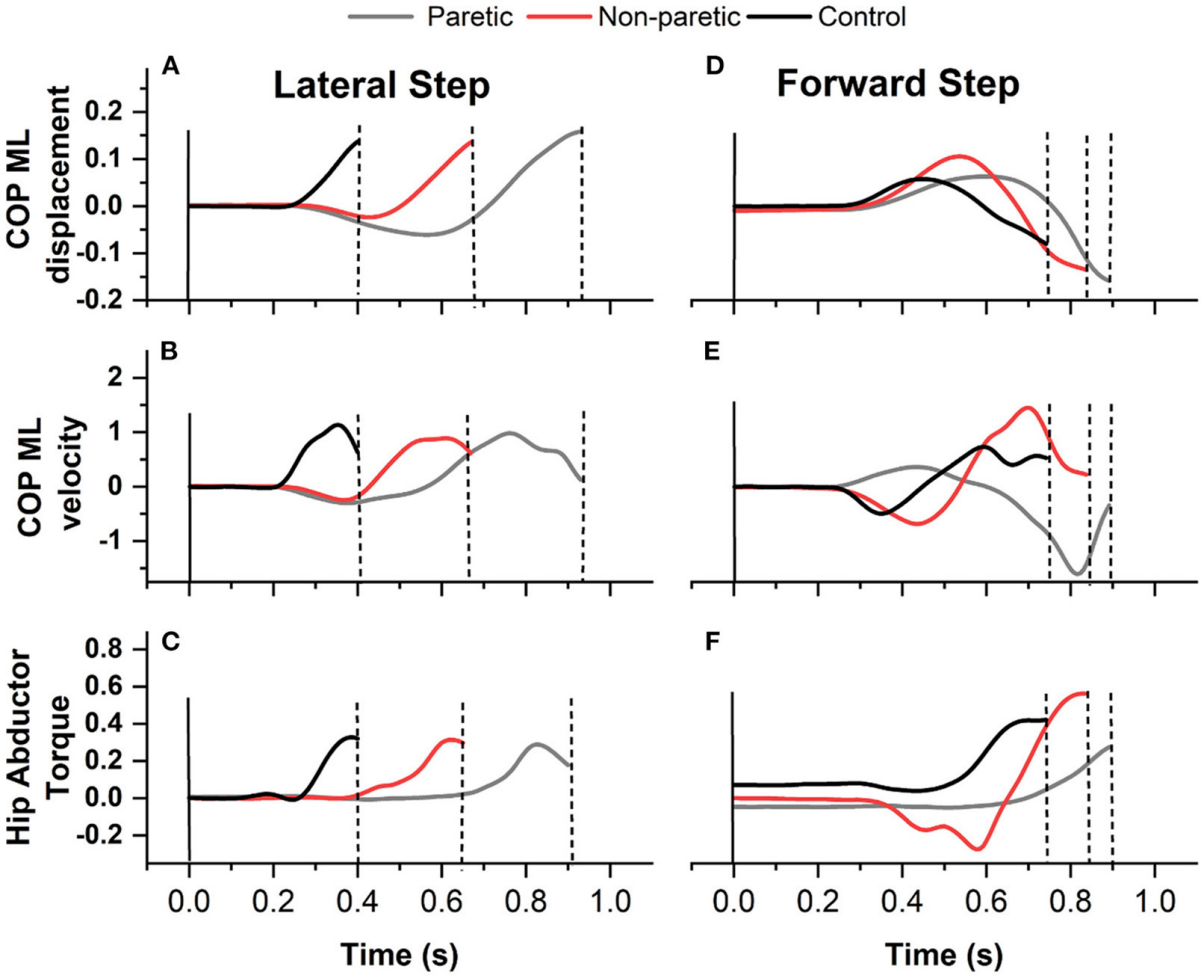
A sample recording of medio lateral center of pressure (ML COP) displacement **(A)** lateral step and **(D)** forward step, ML COP velocity **(B)** lateral step and **(E)** forward step, and hip abductor torque **(C)** lateral step and **(F)** forward step of an individual with stroke stepping with the paretic (gray line) and non paretic leg (red line) and from one control (black line).

The data focused on the WT phase, defined as the interval from the onset of the lateral WT to the instant of the first foot off (14) (Figure 1). Customized Matlab scripts were used to extract the variables during WT: onset time, duration, average mediolateral center of pressure (ML COP) velocity, ML COP displacement, and hip abduction torque (Figure 2), as previously performed elsewhere (6, 18). The WT onset time was defined as 3 SD above the baseline ground reaction force for at least 100 ms relative to the light cue onset time (6). The first foot off was defined as the time when the ground reaction force of the stepping leg was <10 N: The WT duration was the time from the WT onset until the first foot off (6). The ML COP displacement was normalized to the base of support (BOS) width. BOS width was the mediolateral distance between the medial ankle marker between both feet determined prior to the onset of the light cue. The average ML COP velocity was calculated by dividing the ML COP displacement by the WT duration. Inverse dynamics were used to estimate the hip abductor torque. Using the Newton-Euler equations for motion, calculations were performed using the bottom-up approach. The positions of segmental mass centers of gravity, and segmental masses and their moments of inertia were estimated from the anthropometric data of each subject (23). The torque estimates were normalized to the height and weight of the participant.

### Statistical analysis

The values presented are means and standard deviations. The normality of the data was confirmed by using a Shapiro-Wilk test. An independent *t*-test was used to compare demographics and clinical outcome measures between groups (individuals with stroke vs. controls). Non-parametric statistics (Kruskal-Wallis test) were used to compare the cutaneous sensation. The right and left leg of controls were not significantly different, so the values from the right and left leg were averaged, and the average was used for the analyses. A repeated measures analysis of variance (ANOVA) with a between factor (leg) was conducted to assess the effects of step direction (lateral vs. forward) on leg (paretic, non-paretic, and control) for WT characteristics (WT onset time, duration, average ML COP velocity, and ML COP displacement) and hip abductor torque. When a significant main effect was found, *post hoc* comparisons were evaluated using Bonferroni (within comparisons) or Tukey (between comparisons) *post hoc*. Statistical analyses were performed using SPSS v26 (IBM Corp, Armonk, NY), and the significance level was set at *p* ≤ 0.05.

## Results

There were 30 participants, 20 individuals with stroke, and 10 older adults in the control group. There were no significant differences in participant demographics. There were significant between group differences in the TUG (*p* < 0.001), ABC (*p* < 0.001), and the CB&M (*p* < 0.001), Table 1.

**Table 1:** Characteristics of individuals with stroke and control group, expressed as mean **±** standard deviation except for cutaneous sensation expressed as median (25–75 quartile).

	**Controls**	**Individuals with stroke**	
Group			
*n*	10	20	
Gender (Female/Male)	5/5	4/16	
Age (years)	64.8 ± 8.9	61.6 ± 7.4	
Height (m)	1.71 ± 9.3	1.73 ± 0.13	
Weight (kg)	78.5 ± 15.3	91.1 ± 22.7	
Body Mass Index (kg/m^2^)	26.7 ± 5.2	30.3 ± 6.9	
Time Post Stroke (years)	–	8.0 ± 10.0	
Side of Paresis (Right/Left)	–	6/14	
Type of Stroke			
Ischemic		8	
Hemorrhagic		5	
Unspecified		7	
TUG (s)	7.1 ± 1.1	14.0 ± 8.8^*^	
ABC (/100)	95.6 ± 5.6	77.2 ± 12.9^*^	
CB&M Scale (/96)	73 ± 12.2	37.4 ± 13.0^*^	
CMSA (Leg+Foot)	–	9.2 ± 3.18	
	Control	Paretic leg	Non-paretic leg
Cutaneous Sensation	3.65 (3.51–3.92)	4.17 (3.61–4.56)	4.08 (3.22–4.31)

Symbol (*) indicate group differences, p ≤ 0.05.

### Weight transfer characteristics

#### WT onset time

**Figure 3:**
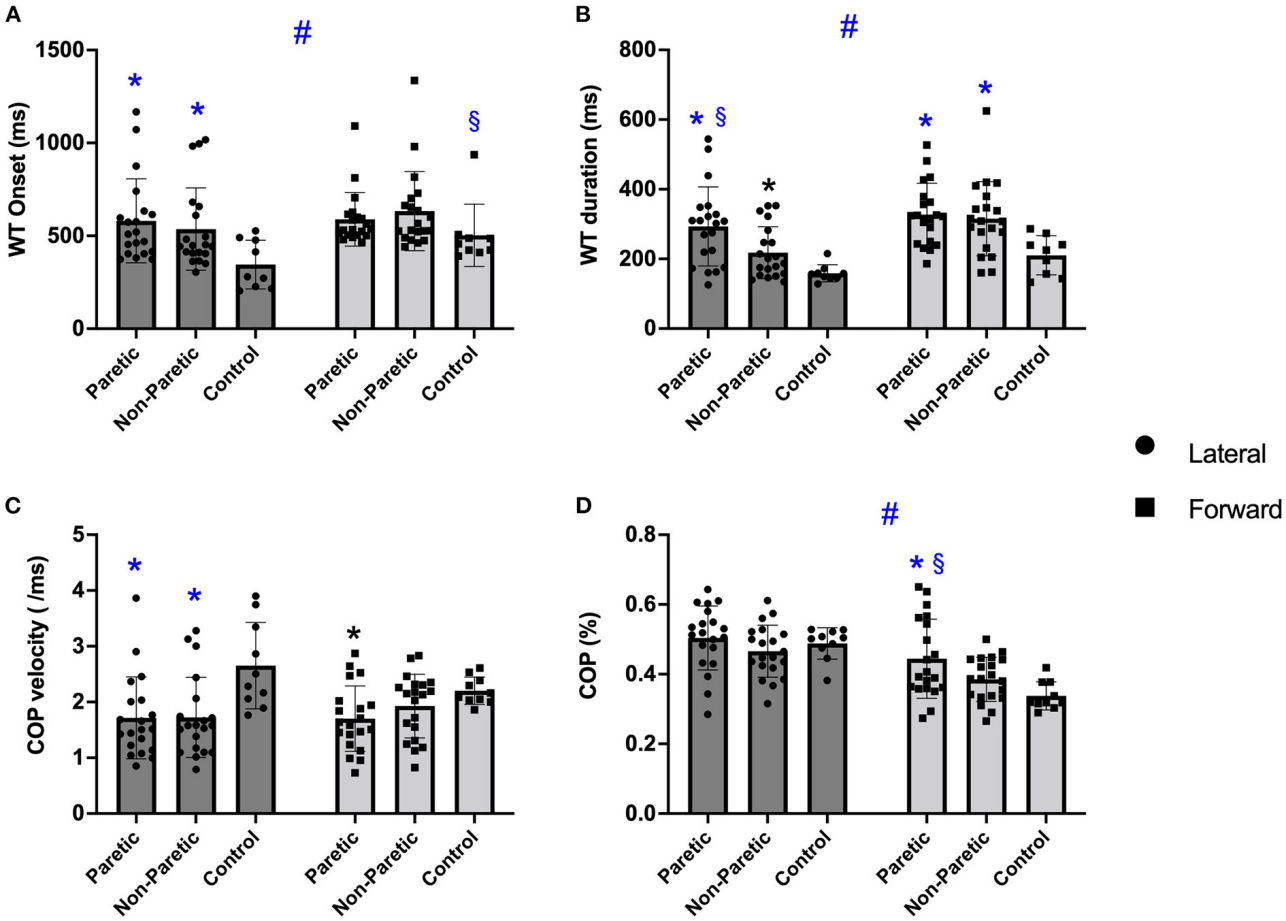
The boxplots of the weight transfer (WT) onset **(A)**, WT duration **(B)**, center of pressure (COP) velocity **(C)**, and COP mediolateral displacement **(D)** before the voluntary lateral and forward choice reaction step between paretic, non-paretic, control groups. Symbols indicate: ^#^*p* < 0.001 different between step direction (forward vs. lateral); ^*^p < 0.005 different from control leg within the same step direction; ^§^*p* < 0.005 different from non-paretic leg within the same step direction.

There was a main effect for step direction [*F*_(1, 46)_ = 13.577; *P* < 0.001], an interaction (step direction by leg) (*P* = 0.046), and a between leg differences (*p* = 0.028). WT onset time was faster before a lateral than the forward step (*p* < 0.001). The paretic and non-paretic legs were slower initiating the WT onset than the control group (*p* ≤ 0.015) before the lateral steps. In comparison, only the non-paretic leg was slower than controls before the forward step (*p* ≥ 0.076), Figure 3A. Overall, the difference between legs showed that the paretic and non-paretic legs were slower to initiate the WT than the control group (*p* ≤ 0.039).

#### WT duration

There was a significant main effect of step direction [*F*_(1, 46)_ = 27.747; *P* < 0.001], a significant step direction by leg interaction (*p* = 0.028) and leg differences (*p* < 0.001). WT duration was quicker before the lateral than the forward step (*P* < 0.001), with the paretic and non-paretic leg taking longer than controls in forward and lateral direction (*p* < 0.001), and the paretic leg taking longer than the non-paretic leg in the lateral direction (*p* ≤ 0.003), Figure 3B. Overall, the controls showed a faster WT duration than the paretic and non-paretic leg (*p* ≤ 0.023).

#### COP velocity

There was no difference between step direction [*F*_(1, 46)_ = 1.003; *p* = 0.322], but there was an interaction (*p* = 0.020) and a difference between legs (*p* = 0.007), Figure 3C. Overall, paretic and non-paretic legs were slower than control (*p* ≤ 0.025). Before the lateral direction, the paretic and non-paretic legs were slower than controls (*p* ≤ 0.002), while before the forward direction, only the paretic leg was slower than controls (*p* = 0.019).

#### ML COP displacement

There was a significant main effect of step direction [*F*_(1, 46)_ = 77.429; *p* < 0.001], a significant step direction by leg interaction (*p* = 0.010) and leg differences (*p* < 0.045) (Figure 3D). The ML COP was greater during lateral than the forward step direction (*p* < 0.001). Additionally, the paretic leg ML COP displacement was greater than the non-paretic leg and controls before a forward step (*p* ≤ 0.039). Although there was a difference between legs, *post hoc* did not identify differences (*p* ≥ 0.084).

### Torque production during the weight transfer

#### Stance leg

**Figure 4:**
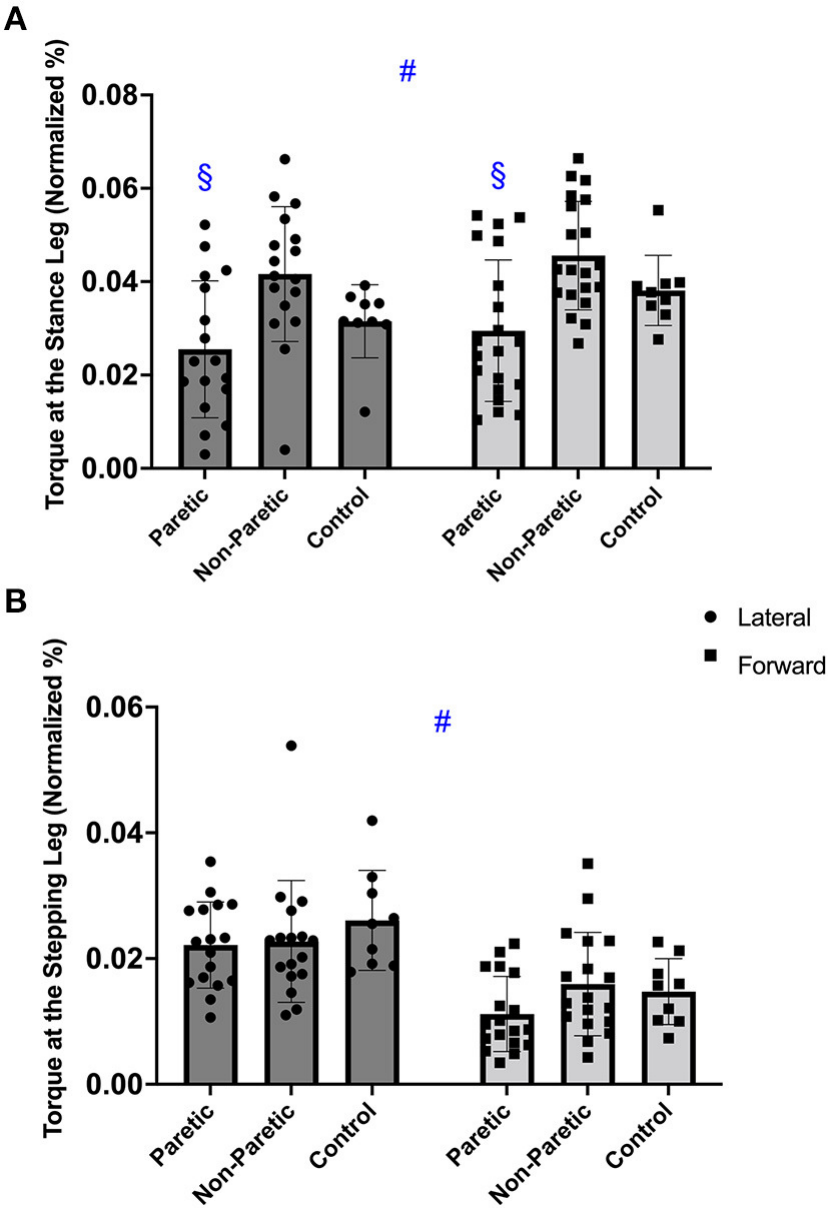
The hip abductor torque in the stance **(A)**, and stepping leg **(B)**, before the voluntary lateral and forward choice reaction step between paretic, non-paretic, and control leg. Symbols indicate: ^#^*p* < 0.001 different between step direction (forward vs. lateral); ^§^*p* < 0.05 different from non-paretic leg within the same step direction.

There was a significant main effect of step direction [*F*_(1, 46)_ = 12.322; *p* < 0.001], with no interaction (*p* = 0.820), but between leg differences (*p* = 0.003). Overall, hip abduction torque of the stance leg was greater before a forward compared to a lateral step (*p* < 0.001). For step direction, *post hoc* demonstrated the paretic leg torque was reduced compared to the non-paretic leg before the lateral and forward steps (*p* ≤ 0.014). Additionally, the paretic and non-paretic leg, had greater torque values before a forward than lateral step (*p* ≤ 0.029), Figure 4A. Overall, the between leg differences were found only between paretic and non-paretic leg (*p* = 0.002).

#### Stepping leg

There was a significant main effect of step direction [*F*_(1, 46)_ = 31.619; *p* < 0.001], with no interaction (*p* = 0.171) or between leg differences (*p* = 0.262). There was greater hip abduction torque in the stepping leg before the lateral compared to the forward step (*p* < 0.001), Figure 4B.

## Discussion

The present study compared the characteristics of the weight transfer phase (onset, duration, ML COP velocity, and ML COP displacement) and hip abductor torque production preceding a lateral and forward voluntary step between individuals with stroke (paretic and non-paretic leg) and controls. Our main finds were: (1) the weight transfer before a lateral step was shorter and initiated earlier, with a larger mediolateral COP displacement and greater hip abductor torque in the stepping leg than the forward step, (2) the weight transfer before the lateral step took longer to initiate and was slower to execute in individuals with stroke regardless of the leg, (3) there was greater hip abductor torque produced in the stance leg before a forward step than a lateral step, and (4) the weight transfer before the forward step had more differences in the paretic than the non-paretic leg. Previous studies have focused on the performance of the stepping leg showing impaired spatiotemporal stepping characteristics. We believe this is the first study to examine the weight transfer characteristics and hip abduction torques before the step. The findings are important for understanding the directional differences in balance control between individuals with stroke and controls. They may also be necessary for developing programs to reduce falls in this population.

### Forward vs. lateral direction

Interestingly, regardless of the leg used (paretic, non-paretic, control), the weight transfer before a lateral step was initiated faster, had a shorter weight transfer duration, resulted in a greater ML COP displacement, and generated a greater hip abduction torque of the stepping leg than a forward step. Before a forward step, a greater hip abduction torque was produced in the stance leg compared to the lateral step. This information reveals an important characteristic of stepping. Although there are differences between groups (individuals with stroke vs. controls), the WT characteristics between the different directions has a similar overall pattern across populations. Thus, weight transfer preceding a lateral step has intrinsic differences compared to forward stepping. The quick weight transfer before the lateral step may present problems for those individuals with stroke since their movements tend to be slower with reduced activation of the lower extremity muscles (24–26). The findings in this study indicate the importance and difficulty of weight transfer before the lateral step given the inherent differences from the forward step. Thus, training to increase the movement speed in individuals with stroke may be necessary for weight transfer control when stepping laterally.

The WT differences between step directions presented here may be explained by different factors. For example, independent of step direction, a step will be preceded by a lateral transfer of bodyweight toward the leg that will support the body during the stance. Thus, the body's center of mass moves toward the stance leg while the stepping leg is free to move. Considering the stepping leg may require greater hip abduction during the lateral step to push the foot off the ground and project the leg laterally, it is reasonable to expect a higher hip abduction torque during a lateral step than the forward step. Conversely, the stance leg had a greater hip abduction torque during the forward step. The longer weight transfer duration before the forward step may allow a greater time for generating torque of the stance leg and may be important for progressing the center of mass forward. Additionally, potentially moving into a smaller base of support for the forward compared to the lateral step may require more preparation. This might happen due to the necessity to move the stepping leg medially before moving forward, while for the lateral step, the leg mainly moves laterally before the step occurs. Therefore, when stepping forward into a smaller base of support greater hip abduction torque may be required to stabilize the stance leg before the step occurs. Since our aim was not to explain the possible differences in hip torque we found here, future studies should further investigate which kinematic variables may influence torque production before the step. This information might provide a further understanding of the factors that influence the ability to step in different directions.

### Individuals with stroke vs. controls

We showed for the first time, individuals with stroke are slower to initiate the transfer of weight compared to individuals. Overall (lateral and forward direction), individuals with stroke, paretic and non-paretic leg, were approximately 48% slower initiating weight transfer, took 89% longer to transfer the weight and 35% slower executing the weight transfer than controls. Along these lines, a study demonstrated that step initiation time took 93% longer in individuals with stroke before forward/backward voluntary steps than controls (27). It is not surprising since individuals with stroke have neural impairments that might affect motor behavior by decreasing the ability to perform a specific motor task (28, 29). Additionally, individuals with stroke also took more time to perform the weight transfer regardless of the step directions, which might be due to different motor planning. A previous study has shown that individuals with stroke may need more time to plan a voluntary step (30) which would explain the differences between individuals with stroke and controls. The slower response of the individuals with stroke compared to controls, and also between paretic and non-paretic leg, may be related to a reduced capacity to activate the muscles needed to transfer weight before the lateral step. Previous research showed that the rate of activation of the hip abductors and adductors muscles influence the time of the weight transfer preceding a voluntary lateral step in older adults (7); hence, individuals with stroke may present a reduced rate of activation of the hip abductors and adductors muscles compared to controls. Moreover, it is possible that a reduced motor cortex excitability (31) and loss of motor units (32, 33) may also affect the ability to prepare the body for stepping.

There were no significant differences in torque production (stance or stepping leg) between individuals with stroke and controls. In contrast, in a different context, other studies demonstrated that individuals with stroke have a lower capacity to produce hip abduction torque (measured at isokinetic dynamometer) than matched controls (9, 34). Thus, torque values measure in-task (e.g., stepping) may provide different outcomes compared to measurements performed outside the task (e.g., isometric test) when comparing individuals with stroke and controls. Although, differences were found between the paretic and non-paretic, the non-paretic leg had an overall hip abduction torque up to ~44% higher than the paretic leg. A study showed that the non-paretic leg may have a negative influence on the paretic leg by decreacreasing the performance of the paretic leg during walking (35), which could impact on step performance. In addition to that, previous research found individuals with stroke had reduced weight bearing on the paretic side (36), which could affect the ability to produce torque in the stance leg during a voluntary step (both directions). However, we controlled the weight bearing in the present study to assure equal weight distribution between paretic and non-paretic legs. Thus, it is possible the reduced velocity demonstrated by the paretic leg (Figure 2), may lead to a lower torque production, helping to explain the differences in torque between legs. Moreover, the ability to produce torque is dependent on the ability to activate the muscles involved in the task (37). For instance, previous research demonstrated impaired muscle activation in the muscles of the paretic leg, while the non-paretic leg had altered muscle activation as a result of compensatory strategies during a forward step (38), which may also help to explain the differences in torque we demonstrated here. Yet, as mentioned above, people individuals with stroke may have a reduced motor cortex excitability and loss of motor unis (31–33) which may lead to an overall motor impairment in the paretic leg (39) which would affect the ability of the paretic leg to perform a step.

Individuals with stroke were slower (COP velocity) during the weight transfer, which also helps to explain the above-mentioned differences between groups. As pointed out above, the neural impairment associated with stroke (28, 29, 31, 33) possibly affects the ability to react quickly and move the lower limbs during a motor task. Furthermore, a similar motor planning, regardless of the leg stepping, may also explain the differences between the individuals with stroke and controls we found in this study (30). Considering motor planning is the integration of sensory afferent information responsible for the position of the limbs (30, 40), this might directly affect the performance during the WT (weight transfer onset, duration, ML COP velocity and ML COP displacement). Indeed, researchers have shown that individuals with stroke may need more time to plan a voluntary step (30), which would explain the differences in WT characteristics between individuals with stroke and controls in the present study.

Nonetheless, a stroke can have an impact on brain function (41), which may reduce cortical excitability (42, 43) and impact balance control (44). Although we did not investigate here the differences in cortical excitability (individuals with stroke vs. controls), it is possible that the impairment of the individuals with stroke we presented might be connected with a decreased brain function. For example, a review pointed out that brain structure (e.g., cerebellum, brainstem) is associated with dynamic balance and leads to a balance disorder (44). Thus, exploring the motor cortex excitability in relation to the WT would be important for future studies.

### Study limitations and relevance

The interpretation of this investigation should be made taking into consideration its limitations. The participants of the present study performed voluntary steps with no external perturbations, and half of all falls an external perturbation is present (e.g., tripping on an obstacle). Yet, understanding a voluntary step is of fundamental importance for individuals with stroke since they present an explicit motor limitation due to their stroke and often limit their ability to step. Moreover, although we investigated the differences between lateral and forward stepping, it would be important to investigate the backward step since many falls also occur in this direction (45). The present study has important implications for health care professionals for understanding directional vulnerability among individuals with stroke. Since our results showed that the transfer of weight preceding a lateral step appears to require a quicker movement than the forward step, the risk of falling may be greater in during lateral stepping. Our results also indicate that a reduced hip abduction torque capacity may place individuals with stroke at risk for falls. Future studies may want to explore how hip abduction neuromuscular activation would contribute to weight transfer across different step directions since individuals with stroke often present a neural impairment. Nonetheless, further understanding if a quicker weight transfer and stronger hip abduction torque can reduce falls among individuals with stroke, would advance training prescription to reduce falls in this population.

## Conclusion

In conclusion, we demonstrated that the weight transfer characteristics and the hip abduction torque during weight transfer differ between step directions (lateral vs. forward) and also between individuals with stroke and controls. Therefore, reduced hip abductor strength may decrease the ability to perform lateral and forward steps in individuals with stroke, which may put this population at risk of falls.

## Data availability statement

The raw data supporting the conclusions of this article will be made available by the authors, without undue reservation.

## Ethics statement

The studies involving human participants were reviewed and approved by Institutional Review Board of the University of Maryland, Baltimore. The patients/participants provided their written informed consent to participate in this study.

## Author contributions

VG performed data collection. All the other parts of the process were equally performed by both authors. All authors contributed to the article and approved the submitted version.

## Funding

This study was developed under a grant from the National Institute on Disability, Independent Living, and Rehabilitation Research (NIDILRR) (H133P100014 and H133F140027). NIDILRR is a center within the Administration for Community Living (ACL), Department of Health and Human Services (HHS). This study was also supported by the American Heart Association (14CRP19880025) and the National Institute on Aging Claude D. Pepper Older Americans Independence Center, P30-AG028747.

## Conflict of interest

The authors declare that the research was conducted in the absence of any commercial or financial relationships that could be construed as a potential conflict of interest.

## Publisher's note

All claims expressed in this article are solely those of the authors and do not necessarily represent those of their affiliated organizations, or those of the publisher, the editors and the reviewers. Any product that may be evaluated in this article, or claim that may be made by its manufacturer, is not guaranteed or endorsed by the publisher.

## Disclaimer

The contents of this publication do not necessarily represent the policy of NIDILRR, ACL, HHS, and you should not assume endorsement by the federal government.

## Abbreviations

ABC: Activities-specific Balance Confidence
CB&M: Community Balance &
Mobility
CMSA: The Chedoke McMaster Stroke Assessment Impairment Inventory
COP: center of pressure
ML: mediolateral
TUG: Timed Up and Go
WT: weight transfer.
